# P2X4 receptors as the dynamic regulators of auditory sensory cell activity: a potential new mechanism for protecting hearing?

**DOI:** 10.1007/s11302-026-10130-0

**Published:** 2026-01-22

**Authors:** Alizée Fisher-Ridoux, Haruna Suzuki-Kerr

**Affiliations:** 1 Department of Physiology, The University of Auckland, Auckland, New Zealand; 2 Eisdell Moore Centre, The University of Auckland, Auckland, New Zealand; 3 School of Medicine, The University of Auckland, Auckland, New Zealand

**Keywords:** Cochlea, Purinergic signalling, P2X4 receptor, Acoustic trauma, Mice

## Article summary

In the cochlea, our peripheral organ for hearing, outer hair cells (OHCs) function as specialised sensory amplifiers that can enhance cochlear sensitivity. The underlying mechanisms that fine-tune such function of OHCs are still poorly understood. The publication by Riffault et al. [1] in the *Journal of Neuroscience* showed that the purinergic signalling mediated by P2X4 receptors plays a central role in modulating OHC activity, potentially protecting OHCs from acoustic trauma and regulating hearing sensitivity in mice [1].

The P2X4 receptor is an ionotropic, ATP-gated cation channel, previously shown to be expressed in the rat [2] and guinea pig [3] cochleae. In this study, the authors embarked on confirming the expression of P2X4 receptors in the mouse cochlea using knock-in mice, P2X4mCherryIN^Exc/Exc^, in the C57BL/6J background. In this mouse model, mCherry is expressed constitutively in the *p2rx4* locus, and the construct is referred to in the article as “*P2X4mCherryIN signal*”. As the C57BL/6J strain of mice has a genetic predisposition to sensorineural hearing loss by 3 months of age, characterisation of these mice was restricted to the age of younger than 60 days old (P60). A strong P2X4mCherryIN signal was observed in the three rows of OHCs in the organ of Corti in both high- to low-frequency regions of the cochlea. At the subcellular level, the P2X4mCherryIN signal was largely concentrated under the cuticular plate (i.e. the actin-rich structure found in the apical part of the OHCs), where the “Hensen body”, an area rich in endoplasmic reticulum and lysosomes, is found. Lysotracker staining suggested an association between P2X4 receptors and the endolysosomal compartments in these regions. P2X4CherryIN signal was also found at the plasma membrane of the basal compartment of OHCs, where OHCs make post-synaptic connections with the medial olivocochlear (MOC) efferent fibres expressing choline acetyltransferase (ChAT).

They then investigated the functional consequence of the P2X4 receptor deletion by generating global P2X4KO and two separate conditional knockout mice: Myo15-Cre^+/−^;P2X4KO^fl/fl^ mice in which the P2X4 receptor is missing from OHCs and inner hair cells (IHCs), and Synapsin1-Cre^+/−^:P2X4^fl/fl^, in which the P2X4 receptor is missing from neurons. They found that P2X4KO mice did not show significant anatomical alteration in the organisation of OHCs, IHCs, or auditory fibres. However, P2X4KO mice at P60 exhibited a significant decrease in click-evoked auditory brainstem (ABR) latencies and thresholds compared to the control group, suggesting an increase in auditory sensitivity in these mice. A similar observation was made in Myo15-Cre^+/−^;P2X4^fl/fl^ mice, but not in Synapsin1-Cre^+/−^:P2X4^fl/fl^. Hence, the observed hypersensitivity of the P2X4-deficient cochlea was attributed to OHC changes. Furthermore, a significant increase in the amplitude of Distortion Product Otoacoustic Emissions (DPOAE) was observed in constitutive P2X4KO and Myo15-Cre^+/−^;P2X4^fl/fl^ mice, but not in Synapsin1-Cre^+/−^:P2X4^fl/fl^. As DPOAE measures OHC activation, these results support a role for P2X4 receptors in cochlear hypersensitivity at the OHC level.

Riffault et al. [1] hypothesised that P2X4 receptors may be regulating the activity of OHCs by modulating the MOC efferent input. The MOC pathway represents one of the efferent innervations to the cochlea. MOC neurons in the superior olivary complex extend efferent projections to the cochlea, where they form synapses on OHCs. The MOC pathway has been implicated in auditory sensitivity turning and protection against acoustic injury [4]. In the MOC pathway, MOC neurons release acetylcholine to activate α9/α10 nicotinic acetylcholine receptors expressed in the post-synaptic OHCs, which subsequently leads to an influx of Ca^2+^, opening of Ca^2+^-dependent K^+^ channels, OHC hyperpolarisation, and reduction in OHC electromotility [5]. The MOC-mediated suppression of OHCs can be studied by activating the cochlea with sounds in the contralateral ear to the one being measured. The authors assessed the activities of the MOC pathway in P2X4KO and conditional knockout mice by using this methodology. In P2X4KO mice, 50-dB SPL stimuli to the contralateral ear resulted in a reduced suppression of the DPOAE amplitudes compared to control animals; in control mice, DPOAEs were completely suppressed but persisted in P2X4KO mice. A similar reduction of MOC suppression was observed in Myo15-Cre^+/−^;P2X4KO^fl/fl^ conditional knockout mice, but not in Synapsin1-Cre^+/−^:P2X4^fl/fl^. Ex vivo labelling with the MOC efferent marker, ChAT, confirmed that the loss of the P2X4 receptor did not change the density or organisation of the MOC efferent terminals. Collectively, these findings support the involvement of P2X4 receptors for activation of the MOC efferent inhibitory pathway and the resulting suppression of OHC activity under homeostatic conditions.

Finally, Riffault et al. [1] investigated the influence of the P2X4 receptor deficiency on the functional recovery from acoustic trauma. P2X4KO mice were exposed to 95-dB SPL white noise for 12 h and assessed at 24 h to 14 days into the recovery period. Twenty-four hours after the noise exposure, both P2X4KO and control mice showed significant reductions in the DPOAE amplitudes, confirming the temporary drop in the OHC function due to noise. Over the 14-day period, P2X4KO mice showed a faster recovery of DPOAE amplitude and from the temporary threshold shift. The half-time of the temporary threshold shift recovery was ~ 3 days in P2X4KO compared to 6 days in the WT controls. Both groups recovered to similar steady-state levels with comparable levels of permanent threshold shifts. Afferent neuronal function, as assessed via ABR wave I amplitude, remained unchanged between controls and P2X4KO groups at P60. They also found that noise exposure of P2X4CherryIN mice resulted in an increase in cytoplasmic fluorescence intensity within the OHC after 48 h. Taken together, the results suggest that the function of P2X4 receptors and the upregulation of P2X4 receptors may increase inhibition from the MOC pathway and slow the functional recovery of OHCs following noise exposure, which may serve as a protective mechanism to prevent further cochlear damage.

## Commentary

The study by Riffault et al. [1] provides valuable insights into the roles of P2X4 receptors in OHCs and adds to our understanding of how purinergic signalling as a whole contributes to the fine-tuning of the cochlea as the sensitive sound-detection organ.

Purinergic receptors (P2X, P2Y, and adenosine receptors) are broadly expressed in the cochlea [6] and the vestibular system [7]. In the spiral ganglion neurons (SGNs) of the cochlea, P2X3 receptors play a role in the refinement of SGN innervation during development [8]. P2X7 receptors are also expressed in type II SGNs [9], although this is in conflict with a previous study, which showed expression of P2X7 receptors in glial cells in the rat cochlea [9]. The present study demonstrates the expression of P2X4 receptors in mouse OHCs based on the P2X4mCherryIN signal. This finding is consistent with previous reports of the P2X4 receptor in rats [2], which utilizes an anti-P2X4 antibody from Alomone Inc for detection. A similar expression pattern for the P2X4 receptor in the sheep and human cochlea has also been reported using the same antibody [10]. Notably, the P2X4 receptor was predominantly cytoplasmic in nature, a common observation reported in other tissues. For example, the P2X4 receptor has been suggested to regulate Ca^2+^-dependent lysosomal fusion [11–13]. In the unique environment of lysosomal compartments, the P2X4 receptor is thought to be protected from degradation by glycosylation [14], and P2X4 receptor activation is regulated by pH within the lysosomal lumen [11, 15, 16] and the local Ca^2+^ level [17]. It would be interesting to clarify the potential association of the P2X4 receptor in Hensen’s body with super-resolution or electron microscopy and further study its function with electrophysiological experiments. It should be noted that whilst the present study reported that P2X4mCherryIN in inner hair cells (IHC) were transient at P6 and reduced in adult mouse P60, studies in rats [2], sheep, and human [10] suggest robust expression of the P2X4 receptor in adult IHCs. The subtle discrepancy could be due to the difference in methodology or the difference between animal models. Even between related species, the molecular expression can vary, as the example case between marmoset and humans for some of the connexin and pannexin channels [18]. Regardless of the differences, the roles of the P2X4 receptor in IHC would be of strong interest to the auditory research community in the future, as IHCs are responsible for providing the majority of sensory output from the cochlea.

Key observations from Riffault et al. [1] are the increased sensitivity to sounds and an impairment in contralateral noise suppression in P2X4KO mice. The complex function of the MOC pathway and its modulation is not yet fully understood. The authors propose that P2X4 receptors work in synergy with cholinergic neurotransmission, as it is well established that ATP can be co-released with acetylcholine in the central nervous system [19]. P2X4 receptors may contribute by supplementing the Ca^2+^ dynamics mediated by nicotinic acetylcholine receptors in the OHCs [20]. In the broader view, the potential function for the P2X4 receptor in the OHC proposed here adds another mechanism by which ATP regulates the cochlear sensitivity. Another member of the P2X receptor subfamily, the P2X2 receptor, has been reported to be broadly expressed in the rodent cochlea, including OHCs and IHCs, supporting cells, Reissner’s membrane (RM), and spiral ganglion neurons [21–28]. Notably, P2X2 receptors are expressed in the OHC at the tip of stereocilia and along the apical boundary of OHCs [25, 28]. In humans, the pathological variation of the *P2RX2* gene has been identified as the cause of autosomal dominant non-syndromic deafness, DFNA41 [26]. The characteristics of the hearing loss in DFNA41 are progressive hearing loss with highly elevated susceptibility to noise-induced hearing loss [26]. Together with functional studies, proposed roles for P2X2 receptors are to modulate the endocochlear potential and the cochlear partition resistance to influence OHC electromotility and to provide otoprotection [29, 30]. In the light of the present study, the activation of both P2X2 receptors and P2X4 receptors seems to have similar effects on the cochlea, dampening the response at the OHC level and reducing the cochlear sensitivity, albeit via potentially different mechanisms. P2X receptors form trimeric channels as the functional unit in either homo- and heteromeric configurations [22]. Hence, we cannot eliminate the possibility that P2X2 receptors and P2X4 receptors form heteromeric channels in OHC and IHC, although the subcellular localisation seems to differ between P2X2 receptors, which are expressed in the stereocilia and apical membrane [25, 28], whilst P2X4 receptors are primarily localised to synaptic and cytoplasmic regions of hair cells [1, 2].

Collectively, the present publication by Riffault et al. [1] adds to our understanding of purinergic receptors and the P2X receptors working in the cochlea to modulate the sensitivity of the cochlea in response to sounds. Adaptation of cochlear sensitivities may provide otoprotection against excessive noise exposure, which could otherwise result in permanent hearing loss. Further understanding of purinergic signalling in the cochlea may enable future treatment strategies targeting purinergic signalling to prevent permanent hearing loss.

## Data Availability

No datasets were generated or analysed during the current study.
